# Experimental and computational methods to highlight behavioural variations in TonB-dependent transporter expression in *Pseudomonas aeruginosa* versus siderophore concentration

**DOI:** 10.1038/s41598-023-46585-z

**Published:** 2023-11-16

**Authors:** Thibaut Hubert, Morgan Madec, Isabelle J. Schalk

**Affiliations:** 1 CNRS, UMR7242, ESBS, University of Strasbourg, Bld Sébastien Brant, 67412 Illkirch, Strasbourg, France; 2 ICube Laboratory, CNRS, UMR 7357, University of Strasbourg, Bld Sébastien Brant, 67412 Illkirch, Strasbourg, France

**Keywords:** Microbiology, Computational science, Molecular biology, Transcriptional regulatory elements, Iron, Membrane proteins

## Abstract

Iron is a key nutrient for bacterial growth. The source can be either heme or siderophore-Fe complexes. Siderophores are small molecules synthesized by bacteria to scavenge iron from the bacterial environment. The pathogen *Pseudomonas aeruginosa* can express at least 15 different iron uptake pathways and all but one involve a TonB-dependent transporter (TBDT) for the uptake of iron across the outer membrane. Little is known about how bacteria modulate and adapt the expression of their different iron import pathways according to their environment. Here, we have developed fluorescent reporters between the promoter region of genes encoding a TBDT and the fluorescent reporter mCherry. With these constructs, we can follow the expression of TBDTs under different growth conditions. Mathematical modelling of the data obtained showed the transcription and expression of the gene encoding the TBDT PfeA to have a sigmoidal shape, whereas it was logarithmic for the TBDT gene *foxA*. Maximum transcription for *pfeA* was reached in the presence of 3 µM enterobactin, the siderophore recognized by PfeA, whereas the maximum was not reached for *foxA* with 100 µM nocardamine, the siderophore of FoxA.

## Introduction

Iron is a key nutrient for almost all microorganisms. It is required for bacterial metabolism, growth, and survival^1–4^. Paradoxically, this essential nutrient shows extremely low solubility (Ksp = 10^–18^ M) under aerobic conditions and at neutral pH, which severely limits its bioavailability^5^. Consequently, iron limitation is a state that microorganisms very often experience and in many ecosystems, the low availability of iron generates fierce competition between them. To overcome iron restriction, many microorganisms synthesize and secrete siderophores, low molecular weight ligands that have a very high affinity for ferric iron (Fe^3+^)^6^. The biological function of siderophores is to scavenge iron in the environment of bacteria and bring it into the bacterial. In Gram-negative bacteria, the uptake of ferri-siderophore complexes across the outer membrane involves TonB-dependent transporters (TBDT)^7^. The energy necessary for the uptake process of ferri-siderophores by these transporters is provided by the inner membrane proton motive force via the inner membrane protein TonB^8–13^. TonB forms a molecular motor with two other membrane proteins, ExbB and ExbD, that are able to transfer the energy generated by the proton motive force to the TBDT located in the outer membrane. Many X-ray structures of TBDTs have been published^7,14–16^. All are composed of a β-barrel of 22 β-strands interacting with the lipids of the outer membrane and contain very large extracellular loops. The lumen of the barrel is filled by a globular domain called the plug. The N-terminal end of the transporter is periplasmic and contains a 5 to 8 amino acid conserved domain called the TonB box, which is essential for interaction with the TonB protein^8,17–19^. All TBDTs have a binding site localized on the plug composed of residues of the plug and the extracellular loops^7^. After binding of the ferri-siderophore complex to its binding site on the plug domain, the TonB box of the TBDT interacts with the periplasmic part of the TonB protein providing the energy necessary to obtain formation of a channel in the transporter and the uptake of ferri-siderophore complexes through the outer membrane^8,12,13,17–19^. Ferri-enterobactin TBDTs, PfeA in *Pseudomonas aeruginosa* and FepA in *Escherichia coli*, have an additional binding site for ferri-siderophore complexes localized in the extracellular loops of the barrel^15,20^. In this case, ferri-enterobactin likely first binds to this binding site before migrating to the binding site on the plug domain.

*P. aeruginosa* is a ubiquitous microorganism known for its high adaptability to a large range of environmental conditions. It is also an opportunist human pathogen, exhibiting high intrinsic resistance to a broad spectrum of antibiotics. To access iron, *P. aeruginosa* produces two siderophores, pyoverdine and pyochelin, but is also able to use siderophores from other microorganisms in a piracy strategy^21^. The genome of *P. aeruginosa* encodes 35 TBDTs and at least 20 are dedicated to iron import, three for zinc, one for copper, and one for vitamin B12^21,22^. The transcription and expression of the genes encoding TBDTs involved in iron acquisition are all regulated by the bacterial intracellular iron concentration. When iron in the cytoplasm of *P. aeruginosa* cells reaches a threshold concentration, iron binds to the transcriptional regulator Fur and the complex formed represses the transcription of all genes encoding proteins involved in iron homeostasis, including those for TBDTs^23–26^. Transcription of some genes encoding TBDTs are also positively regulated, involving sigma ECF/anti-sigma ECF factors, two-component systems, or AraC-like transcriptional regulators^27–32^. These three different systems can detect the ability of *P. aeruginosa* to scavenge and import specific ferric-siderophore complexes present in its environment and activate transcription of the gene encoding the TBDT able to import the ferri-siderophore detected. This results in an increase in the expression of the TBDT of the ferri-siderophore complex present in the bacterial environment to increase the efficiency of its uptake^33–37^. This often goes hand in hand with repression of the transcription of *fptA*, the gene encoding the TBDT of ferric-pyochelin (one of the two siderophores produced by *P. aeruginosa*).

Until now, the regulation of transcription and expression of genes encoding TBDTs has been investigated only in the presence of its ligand at one or a few concentrations and at a given time of culture, i.e., stopping the culture and analyzing the levels of transcription or expression using techniques such as RT-qPCR or proteomics^30,33–37^. How the transcription of such genes changes and varies during bacterial growth and in the presence of different concentrations of siderophores is not precisely known. Here, we aimed to monitor and model the changes in transcription and expression of TBDTs involved in the acquisition of iron across concentration gradients of siderophores. Accordingly, we investigated the transcription of two genes encoding TBDTs, *pfeA* and *foxA*, throughout the duration of bacterial culture and in the presence of a wide range of concentrations of siderophores. PfeA imports iron via the tricatechol-type siderophore enterobactin (ENT, Fig. S1A), a molecule produced by *Escherichia coli* and *Salmonella typhimurium*^15,33^. Through its three catecholate functions connected to a triserine macrocycle, ENT is the siderophore with the highest known affinity for ferric iron (Ka = 10^49^ M^−1^)^38^. FoxA transports iron complexed to hydroxamate siderophores such as nocardamine (NOCA, Fig. S1B, produced by *Streptomyces wadayamensis* or *Streptomyces parvulus*), a cyclic trihydroxamate molecule also known as desferrioxamine E^16,35^. The affinity for ferric iron of this siderophore is weaker than that of ENT (Ka = 10^32^ M^−1^)^39^. The X-ray structures of these two TBDTs are known and data are available concerning how ferri-ENT and ferri-NOCA interact with their respective binding sites on PfeA and FoxA^15,16^. We followed the expression of these two TBDTs using promoter fusions: the promoter region of one of these two genes was fused to the coding sequence of the fluorescent protein mCherry, used as reporter^40^, and inserted into the genome of *P. aeruginosa* to have a fluorescent reporter strain for each TBDT. We show these fluorescent reporter strains to be promising tools that make it possible to follow the real-time transcription and expression of *pfeA* and *foxA* by fluorescence throughout bacterial growth and in the presence of a wide range of siderophore concentrations. Surprisingly, we did not observe maximum transcription and expression at the same concentrations of siderophores for the two genes. Mathematical modeling was used to precisely characterize the expression levels of these two genes as a function of time and siderophore concentration.

## Results

### Construction of the fluorescent reporter strains prom*pfeA*-mCherry and prom*foxA*-mCherry to follow and investigate PfeA and FoxA expression

**Figure 1:**
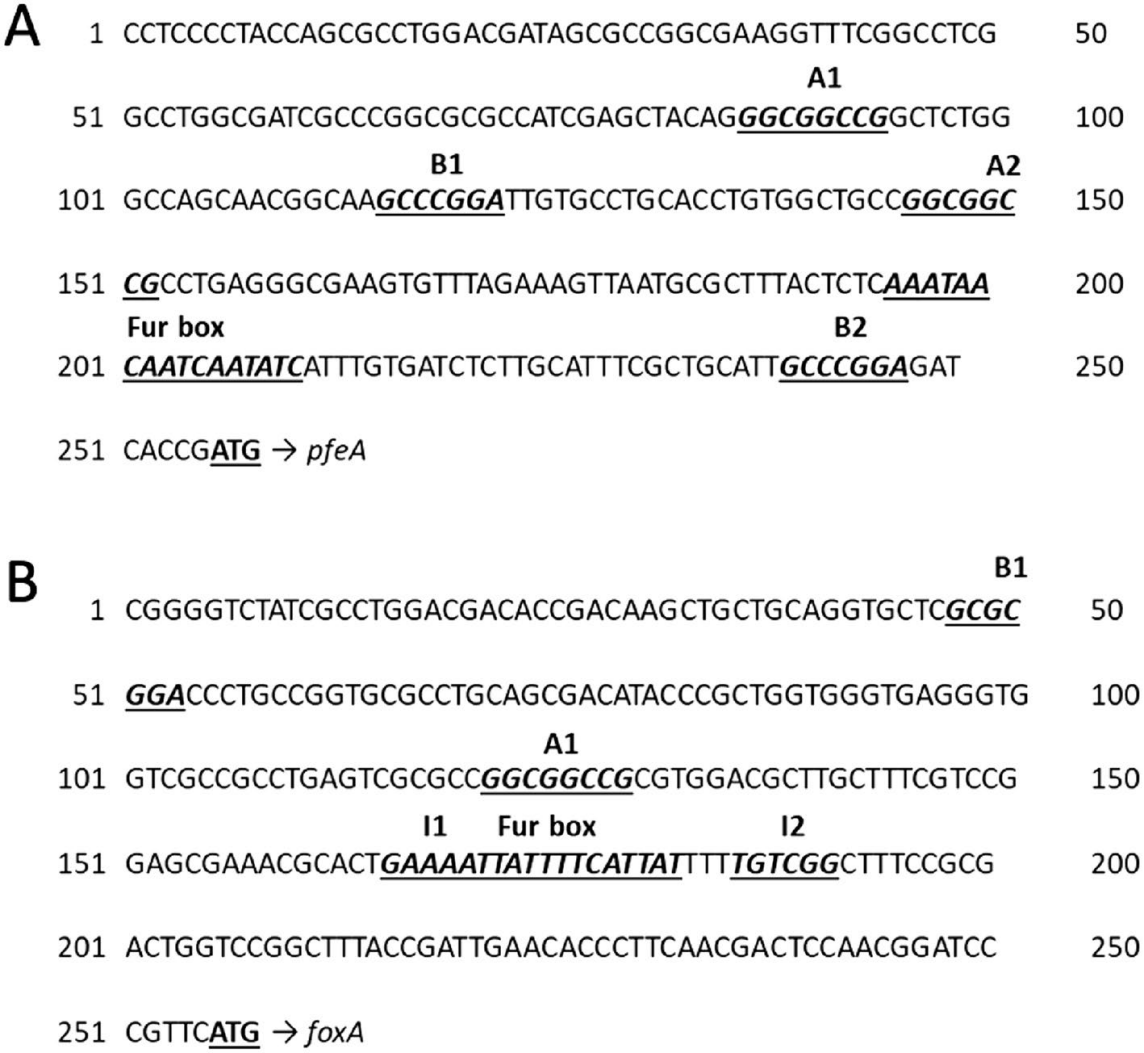
Promoter sequences selected for transcriptional fusion. DNA sequences upstream of the ATG codon of *pfeA* (**A**) and *foxA* (**B**). The Fur box for both genes is highlighted, as are the binding sites of PfeR (A1, A2; B1, B2) for *pfeA* and the predicted binding sites of FoxI (I1, I2) for *foxA*. For *pfeA*, A1: [86–93]; B1: [115–121]; A2: [145–152]; Fur box: [195–211]; B2: [241–247]. For *foxA*, B1: [47–53]; A1: [121–128]; I1: [165–170]; Fur box: [165–182]; I2: [186–191]. The two 255-bp sequences presented here were used as promoter regions to construct the fluorescent reporter strains prom*pfeA*-mCherry and prom*foxA*-mCherry.

We chose to use the TBDTs PfeA and FoxA, involved in iron acquisition by ENT and NOCA, respectively^15,16^, to create a mathematical model of TBDT expression by *P. aeruginosa*. To follow the expression of the *pfeA* and *foxA* genes, we used promoter fusions in which the promoter sequences of the *pfeA* or *foxA* gene were fused to the coding sequence of a reporter that can be easily detected or quantified^40^. In the promoter fusions, the transcription of the reporter gene is controlled by the promoter sequences of the gene of interest, which in turn controls the quantity of mRNA transcribed and, subsequently, that of the protein synthesized. Here, we used the red fluorescent protein mCherry because of its well-documented stability and efficiency as a reporter^41–43^. The promoter regions of *pfeA* and *foxA* both contain a Fur box, involved in transcriptional regulation via the regulator Fur and the bacterial intracellular iron concentration (Fig. 1). *pfeA* transcription is also positively regulated by a two-component system involving the inner membrane sensor PfeS and the transcriptional regulator PfeR^27,44,45^. Consequently, the *pfeA* promoter region also contains a sequence that interacts with PfeR (Fig. 1A). Dean et al. identified the various sequences recognized by PfeR in the promoter sequence of *pfeA*, and called them A1, A2, B1, and B2 (Fig. 1A)^44^. These binding sites consist of pairs of palindromic sequences located upstream of and near the starting codon of *pfeA*. Even if deletion of *pfeR* abolishes *pfeA* transcription and expression^27,31^, the regulation of *pfeA* transcription is certainly more complex since *pfeA* seems also to be regulated by two other transcriptional regulators, PirR and CzcR, both also involved in two-component systems (PirS/PirR and CzcS/CzcR)^46^. The precise mechanisms and interconnection between these three transcriptional regulators involved is not clearly elucidate so far. *foxA* transcription is positively regulated by a sigma factor (FoxI) and its anti-sigma factor (FoxR)^30^, and the promoter region of *foxA* contains the sequences I1 and I2, predicted to be recognized by FoxI (Fig. 1B). Surprisingly, one copy of each palindromic sequence A and B found in the promotor region of *pfeA* is also found in the promotor region of *foxA*. The role they play in the regulation of *foxA* transcription is unknown.

For both genes, we selected a 255-bp sequence directly upstream of the the ATG codon. These two regions, followed by the DNA sequence of mCherry, were inserted into the genome of *P. aeruginosa* between the *glm*S and PA5548 genes and the strains generated are called prom*pfeA*-mCherry and prom*foxA*-mCherry, respectively (Table S1). This site on the chromosome, well conserved between *P. aeruginosa* isolates, has already been used to insert the 4500 bp of the pUC18T mini-Tn7T-Gm vector, derived from the Tn7 transposon^47^. We found that insertion of our sequence in the genome of *P. aeruginosa* did not significantly modify transcription of the *glm*S or *PA5548* genes located next to the insertion locus by RT-qPCR: similar levels of transcription of these two genes were observed in the prom*pfeA*-mCherry and prom*foxA*-mCherry strains grown in the presence of either ENT or NOCA as in wild type PAO1 (Fig. S2). We also checked that the addition of a second promoter in the genome did not interfere with the transcription of *pfeA* and *foxA,* again by RT-qPCR. Indeed, we observed similar levels of *pfeA* and *foxA* transcription in PAO1 as in the prom*pfeA*-mCherry and prom*foxA*-mCherry strains grown in the presence of either ENT or NOCA (Fig. S3).

### Regulation of mCherry transcription by the promoter regions of *pfeA *and *foxA*

**Figure 2:**
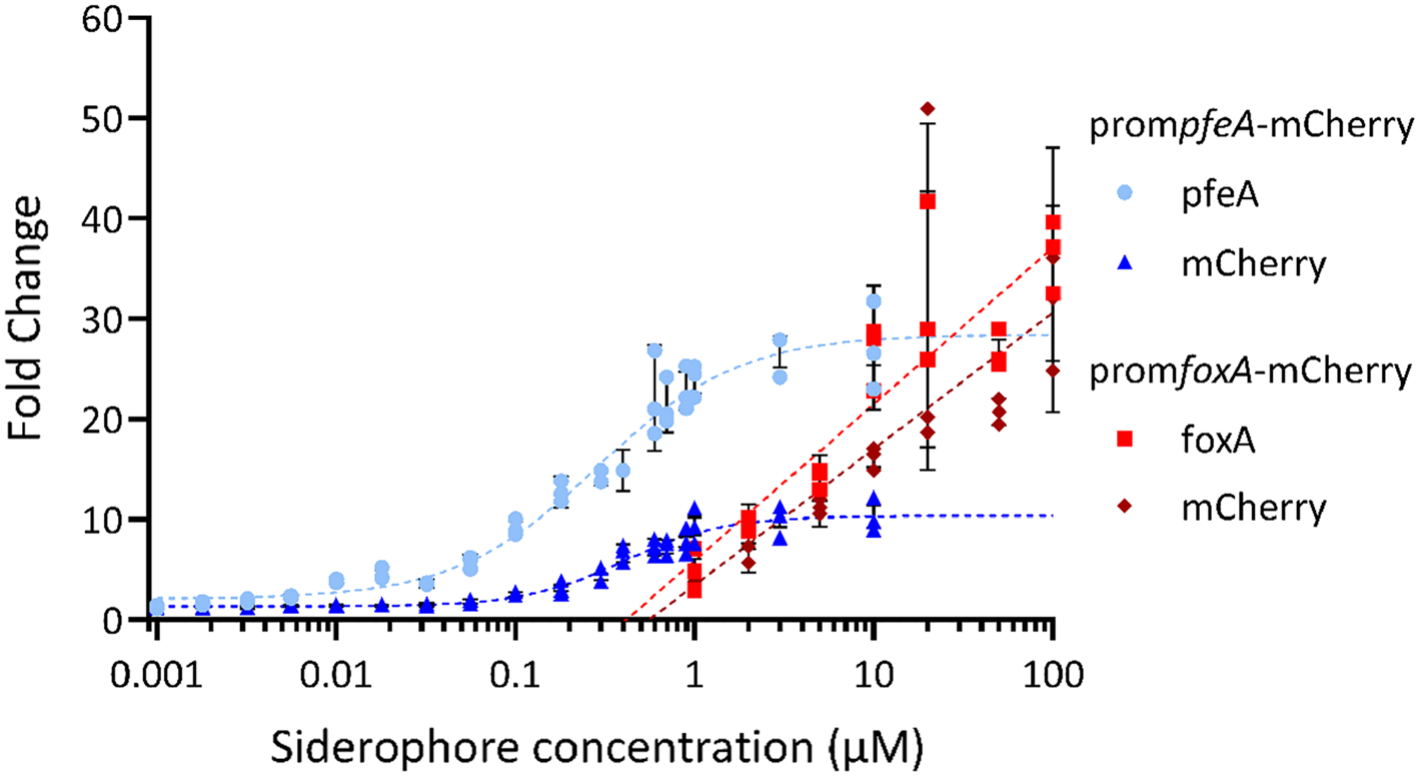
Correlation between *mCherry* and *pfeA* or *foxA* transcription in prom*pfeA*-mCherry and prom*foxA*-mCherry cells in the presence of increasing concentrations of ENT and NOCA, respectively. The *P. aeruginosa* prom*pfeA*-mCherry and prom*foxA*-mCherry strains were grown for 8 h in CAA medium in the absence or presence of increasing concentrations of ENT (0.001 to 10 µM) or NOCA (1 to 100 µM). The transcription of *pfeA* (light blue points) *and mCherry* (dark blue triangles) was followed by RT-qPCR in prom*pfeA*-mCherry and *foxA* (red squares) and *mCherry* (dark red diamonds) in prom*foxA*-mCherry. The results are expressed as the ratio of the values obtained for the growth in the presence of the siderophores to those obtained in their absence. Each concentration of ENT or NOCA was tested using biological triplicates. The error bars represent the standard errors calculated by CFX Maestro™ Software (Bio-Rad). The reference genes used were *clpX* and *rpoD*. The data for prom*pfeA*-mCherry were fitted using the Hill Eq. (1), as the two data sets showed a sigmoid shape. The two data sets for prom*foxA*-mCherry showed a linear shape in the logarithmic domain and, thus, a logarithmic Eq. (2) was used for each fit.

We used a RT-qPCR approach to verify that there is, indeed, a correlation between the level of transcription of the *mCherry* gene and that of *pfeA* or *foxA* in the prom*pfeA*-mCherry and prom*foxA*-mCherry strains, respectively. Such a correlation is essential to use mCherry fluorescence to follow *pfeA* and *foxA* expression in prom*pfeA*-mCherry and prom*foxA*-mCherry. The two strains were grown under iron-restricted conditions (CAA medium) in the presence of increasing concentrations of ENT or NOCA, and the transcription of the *mCherry*, *foxA*, and *pfeA* genes monitored by RT-qPCR (Fig. 2). The mRNA levels of both *pfeA* and *mCherry* increased in the prom*pfeA*-mCherry strain as a function of the ENT concentration with a similar sigmoid shape. Transcription started at approximately 0.1 µM ENT and the maximal level of mRNA synthesis was reached at 3 µM for both genes. As the two data sets showed a sigmoid shape, the data were fitted using the Hill Eq. (1)^48–50^

1 $$H\left(x\right)= {y}_{0}+\left({y}_{max}- {y}_{0}\right)\cdot \frac{{x}^{n}}{{K}^{n}+{x}^{n}},$$

with $H(x)$ as the genetic expression, $x$ the concentration of the siderophore, ${y}_{0}$ the basal genetic expression, ${y}_{max}$ the maximal genetic expression, $K$ the effective affinity, defined as the siderophore concentration needed for the genetic expression to be half of the maximal expression, and $n$ the Hill coefficient, which determines the steepness at the transition between the inactive and active state around $x=K$. We applied the non-linear least-square minimization algorithm to fit the Eq. (1) with the experimental measurements and found the following values for the model parameters: ${y}_{0}$ = 2.06, ${y}_{max}$ = 28.47, $n$ = 1.09, $K$ = 0.29 µM for *pfeA* and ${y}_{0}$ = 1.33, ${y}_{max}$ = 10.37, $n$ = 1.55, and $K$ = 0.40 µM for *mCherry*. However, the transcription levels of the transporter gene *pfeA* were higher than those of *mCherry* for all ENT concentrations tested.

For the prom*foxA*-mCherry strain, the transcription levels of *foxA* and *mCherry* followed the increasing gradient of NOCA concentrations in a similar logarithmic manner, but not as a sigmoid curve as for *pfeA* in the prom*pfeA*-mCherry strain. The maximum transcription for both genes was apparently not reached at 100 µM NOCA (Fig. 2). A piecewise linear equation in the logarithmic domain (2) was used to model this behavior as follows:

2 $$L\left(x\right)=\left\{\begin{array}{l}0, x<{x}_{E}\\ a\cdot {\text{log}}_{10}(\frac{x}{{x}_{E}}), x\ge {x}_{E}\end{array}\right..$$

With $L(x)$ as the genetic expression, $x$ the concentration of the siderophore, $a$ the slope of this logarithmic model, and ${x}_{E}$ the threshold above which the logarithmic behavior starts. We used the same fitting algorithm and found $a$ = 15.58, ${x}_{E}=0.42$ µM for *foxA* and $a$ = 13.58, ${x}_{E}$ = 0.56 µM for *mCherry*. The level of transcription of the transporter gene *foxA* was also higher than that of *mCherry*.

Both the *pfeA* and *mCherry* RT-qPCR fold-change data sets followed a sigmoid-shaped curve, but their Hill equation parameters showed differences. The parameters ${y}_{0}$, $n$, and $K$ from the two equations were relatively close to each other, but ${y}_{max}$ was much higher for *pfeA* than *mCherry*. To quantitatively assess the likelihood of the expression of *pfeA* and *mCherry*, we first normalized the data and then compared them. The method was the following: we (i) normalized the data ($\widehat{{FC}_{pfeA}}$ and $\widehat{{FC}_{mCh}}$) between 0 and 1 and put them together in a single dataset we named *pfeA* + *mCherry*, (ii) fit this dataset with a single Hill equation $\widehat{H}(x)$, and (iii) compared the estimated root mean square error (RMSE) $RMS{E}_{pfeA}$ and $RMS{E}_{mCh}$ between this model, which corresponds to the common trend, and the normalized data of each experimental set taken individually (that for *pfeA* and *mCherry*).

3 $$\widehat{H}\left(x\right)=\frac{{x}^{n}}{{K}^{n}+ {x}^{n}},$$

4 $$\widehat{{FC}_{pfeA}} =\frac{{FC}_{pfeA} -{y}_{0 pfeA}}{{y}_{\mathrm{max}pfeA} -{y}_{0 pfeA}},$$

5 $$\widehat{{FC}_{mCh}} = \frac{{FC}_{mCh} -{y}_{0 mCh}}{{y}_{max mCh} -{y}_{0 mCh}}.$$

**Figure 3:**
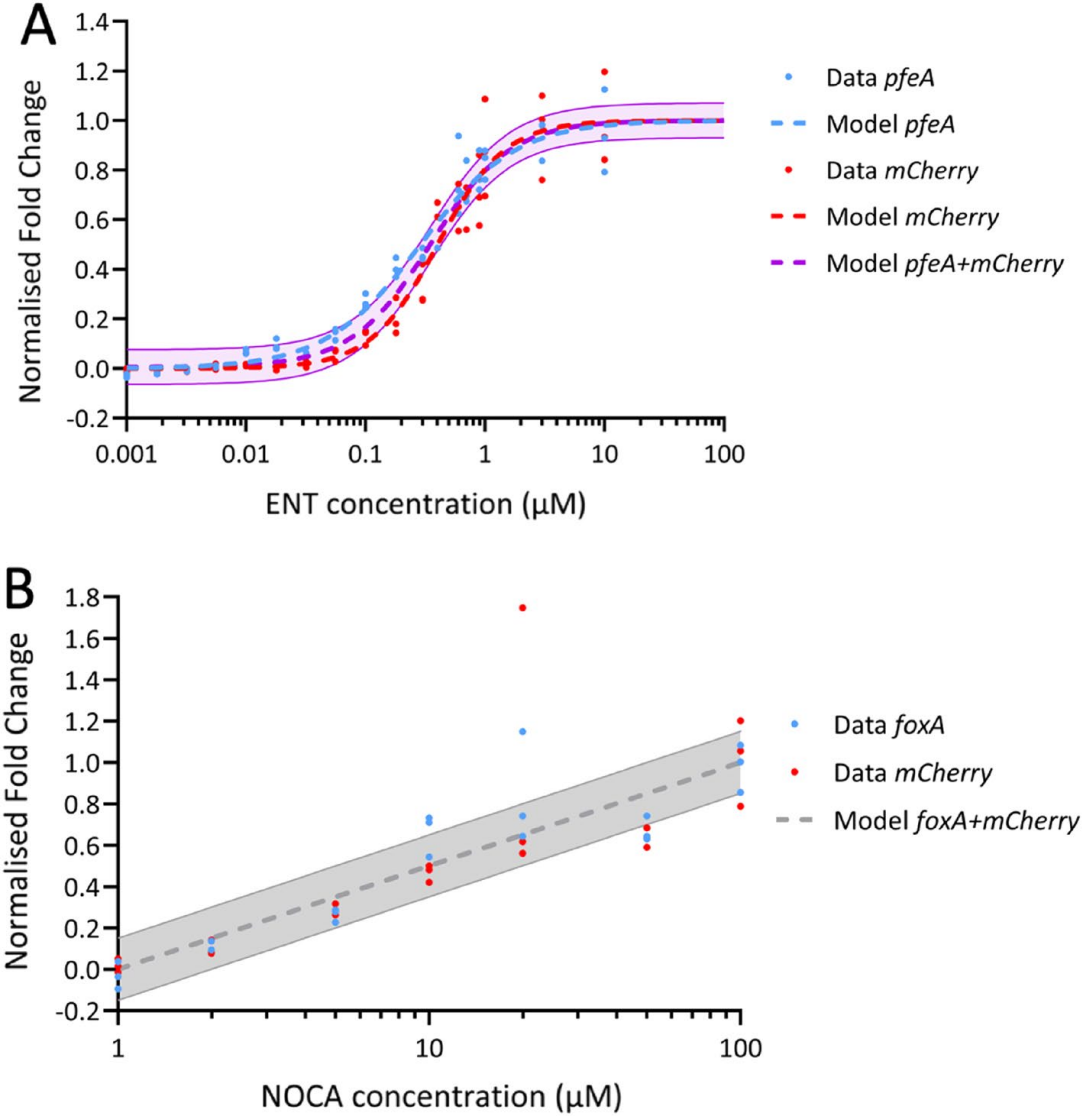
Normalization of *pfeA*, *foxA* and *mCherry* transcription. (**A**) The normalized data sets of RT-qPCR fold changes from the prom*pfeA*-mCherry strain are represented in blue for *pfeA* and in red for *mCherry* (data as dots and the Hill curve as the dashed line). The Hill curve of the *pfeA* + *mCherry* data set, with an error of ± 7%, is represented by the purple dashed line and the transparent purple area. (**B**) The normalized data sets of RT-qPCR fold changes from the prom*foxA*-mCherry strain are represented in blue for *foxA* and in red for *mCherry*. The logarithmic normalized data sets of *foxA*, *mCherry*, and *foxA* + *mCherry* are modeled using the same logarithmic equation, with an error of ± 15% represented as a grey dashed line with a transparent grey area.

The normalized and pooled data sets are presented in Fig. 3A with the fitted Hill curves. We calculated the RMSE between the normalized *pfeA* and *mCherry* whole data sets and the *pfeA* + *mCherry* Hill equation. We found RMSE values of 0.052 and 0.051 for the comparison of the *pfeA* data set versus that of *pfeA* + *mCherry* and the *mCherry* data set versus that of *pfeA* + *mCherry*, respectively. We also calculated the RMSE solely for the transition phase by reducing the range of the dataset on the $y$-axis, first between 10 and 90% of maximal expression and then between 20 and 80% of maximal expression. We found RMSE values of 0.065 and 0.070 for *pfeA* and *mCherry*, respectively, for the first range and 0.070 and 0.071, respectively, for the second. Therefore, the error of our *pfeA* + *mCherry* model is approximately 7%. To obtain an idea of what such an error represents, we calculated the dispersion, which intrinsically exists in the data, by calculating the RMSE between the data and the Hill dataset-specific model for both genes. All RMSE values were of the same order of magnitude, regardless of the domain. Thus, modeling both datasets using a unique model did not appear to introduce any additional error to that intrinsically present in the dataset, suggesting that the model is valid. In conclusion, *pfeA* and *mCherry* behave in the same manner with respect to the siderophore concentration in qRT-PCR.

We normalized the data from prom*foxA*-mCherry using the same method and created the pooled data set *foxA* + *mCherry*. The logarithmic models of the normalized transcriptional expression data of *foxA* and *mCherry* and those of *foxA* + *mCherry* all had the same parameters ($a$ = 0.5 and ${x}_{E}$ = 1 µM, Fig. 3B). We calculated the RMSE between the normalized *foxA* and *mCherry* data sets and the *foxA* + *mCherry* logarithmic equation and found RMSE values of 0.123 and 0.151 for the comparison of the *foxA* data set versus that of *foxA* + *mCherry* and the *mCherry* data set versus that of *foxA* + *mCherry*, respectively. The error of our *foxA* + *mCherry* model is aproximately 15%. Using the same approach as for *pfeA* and *mCherry*, we computed the intrinsic dispersion between the *foxA* or *mCherry* data and the *foxA* + *mCherry* model. The RMSE values between the data and the model were also of the same order of magnitude. The use of a single model for both data sets did not introduce any additional error beyond the intrinsic variability present in the data. Hence, the validity of the model remains unaffected. In conclusion, *foxA* and *mCherry* exhibit similar behavior as a function of the siderophore concentration in qRT-PCR.

Overall, the prom*pfeA*-mCherry and prom*foxA*-mCherry constructs meet all the criteria of a fusion reporter. For both constructs the transcription of *mCherry* follows that of the studied TBDT, demonstrating that *mCherry* transcription and expression are regulated by the promoter region of both TBDTs. However, it should be noted that we observed a higher level of transcription for the genes encoding the TBDT than that of *mCherry* for both constructs. This difference was more pronounced in the case of the fusion reporter carrying the *pfeA* promoter region. Interestingly, the transcription of both the *pfeA* and *foxA* genes appears to not respond in the same way nor with the same efficiency to the presence of their siderophores. Maximum transcription was reached for *pfeA* with 3 µM ENT, whereas the maximum was not reached for *foxA* with 100 µM NOCA.

### mCherry expression modelling in both prom*pfeA*-mCherry and prom*foxA*-mCherry cells

**Figure 4:**
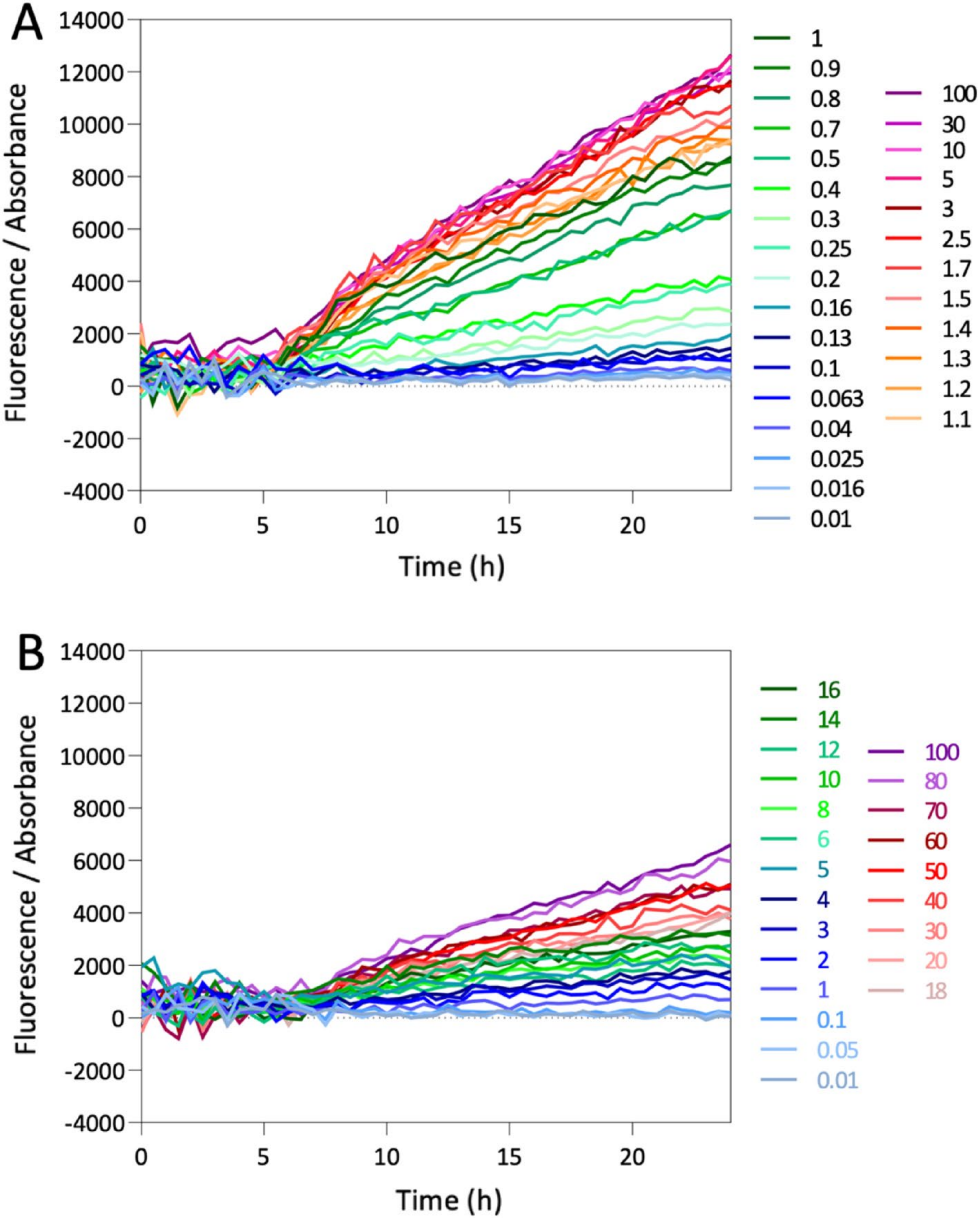
mCherry fluorescence monitored in both prom*pfeA*-mCherry (**A**) and prom*foxA*-mCherry (**B**) strains. prom*pfeA*–mCherry and prom*foxA*–mCherry strains were grown in CAA medium in the absence or presence of increasing concentrations of ENT and NOCA (0.01µM to 100 µM), respectively, and the OD_600 nm_ and fluorescence of mCherry (excitation at 570 nm and emission at 610 nm) monitored. The bacterial fluorescence rate calculated using Eq. (6) is represented for each concentration. Three independent experiments with three technical replicates were performed for each concentration of ENT and NOCA tested (n = 3). Only the means of the three data points are shown and no error bars are shown for convenience.

The transcription kinetics of *mCherry* and *pfeA* in *prompfeA-mCherry* and those of *mCherry* and *foxA* in *promfoxA-mCherry* follow the same dynamics, allowing the two fusion reporters to be used to monitor the expression of the two TBDTs under different growth conditions. Stimulation of *pfeA* or *foxA* transcription and expression lead to an increase in mCherry synthesis and, consequently, in the monitored fluorescence of mCherry. We followed *pfeA* and *foxA* expression by monitoring mCherry fluorescence during prom*pfeA*-mCherry and prom*foxA*-mCherry growth in CAA medium with increasing concentrations of ENT and NOCA. Bacterial growth was monitored by measuring the optical density at 600 nm (OD_600 nm_) and the fluorescence of mCherry at 610 nm (excitation wavelength: 570 nm) as a function of time (Fig. 4A,B). There was no significant increase in fluorescence for either construct at any tested concentration of ENT or NOCA for the first 6 h of culture. For prom*pfeA*-mCherry, we observed an increase in mCherry fluorescence during bacterial growth for concentrations of ENT of 0.1 µM or higher, with maximum fluorescence obtained from 3 µM of ENT and higher (Fig. 4A). For prom*foxA*-mCherry, the monitored fluorescence of mCherry increased with increasing concentrations of NOCA and no saturation was observed (Fig. 4B).

For each condition, the fluorescence data were treated as follows: the fluorescence data of the control (without siderophore) was first subtracted from the fluorescence of the condition of interest. Then, for each condition, the resulting fluorescence was divided by the OD_600 nm_ (${Absorbance}_{condition}$), which represents the density of the bacteria in the sample (6). Within a factor of one, the result can be considered to be the fluorescence signal emitted by a single bacterium.

6 $$({Fluorescence}_{condition}-{Fluorescence}_{control})/{Absorbance}_{condition},$$

### Rate of mCherry synthesis

To characterize all the curves of the fluorescence data shown in Fig. 4, we used the synthesis Eq. (7) for each condition. We modeled the transient evolution of the fluorescence signal using two assumptions: (i) the fluorescence signal is directly proportional to the concentration of mCherry: $F=k\cdot P$, where $F$ is the fluorescence signal and $P$ the concentration of mCherry, and (ii) the temporal evolution of the concentration of mCherry can be modeled by a first-order differential Eq. (7) involving the gene expression $\beta$ (in µM/h) and the degradation rate of mCherry $d$ (in h^−1^).

7 $$\frac{dP}{dt}=\beta -d\cdot P.$$

The solution of this differential equation is given in (8)

8 $$P\left(t\right)= {P}_{\infty }\cdot \left(1- {e}^{\left(- \frac{t- {t}_{0}}{\tau }\right)}\right),$$

where ${P}_{\infty }$ is the concentration of mCherry at the steady state (concentration reached after an infinite time), ${t}_{0}$ represents the lag time or adaptation time (time between the start of culture and the beginning of mCherry synthesis, which also depends on the fluorescence detection threshold of the analytic instrument), and $\tau$ is the characteristic time, which is equal to $2.2/d$, and corresponds to the time required to reach 63% of ${P}_{\infty }$.

According to Eq. (8), at steady state, we can state that

9 $$\beta = \frac{{P}_{\infty }}{\tau }.$$

**Figure 5:**
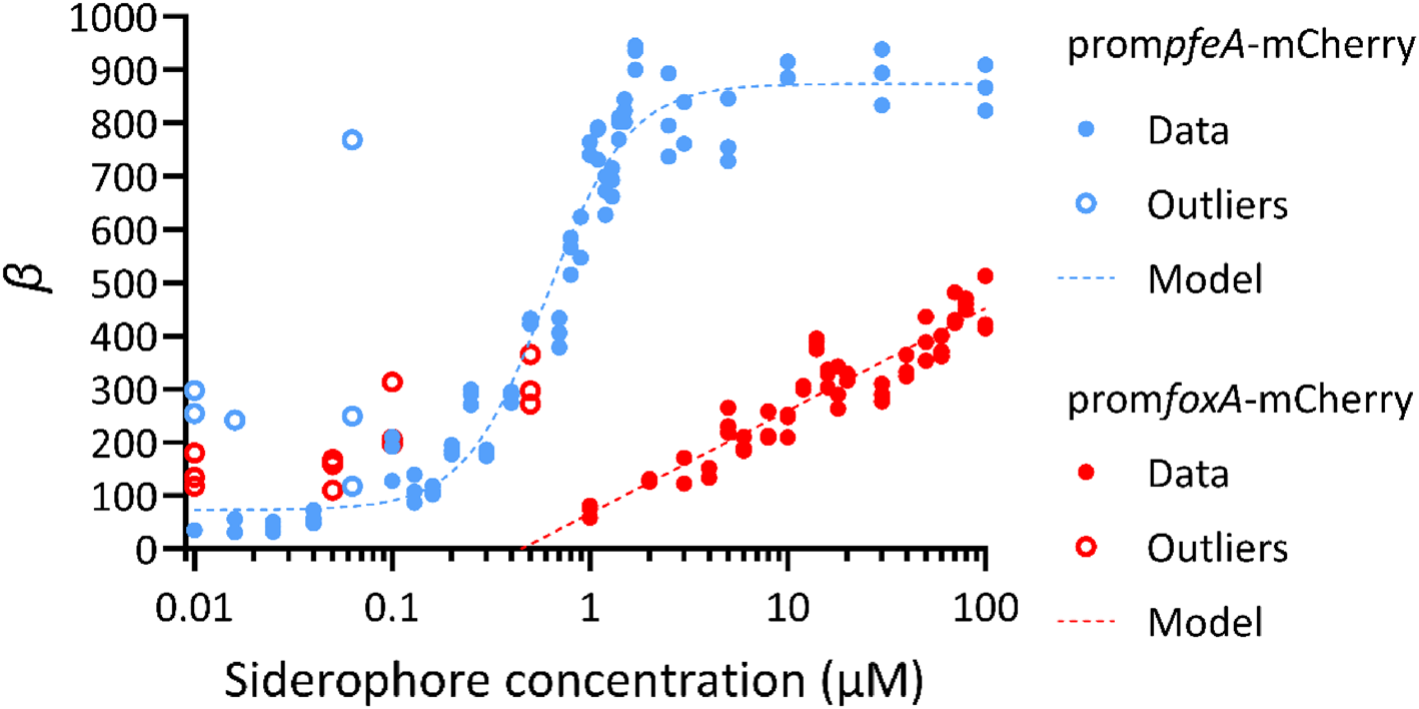
Fold change in *mCherry* synthesis in prom*pfeA*-mCherry and prom*foxA*-mCherry cells grown in iron-restricted conditions and in the presence of increasing concentrations of ENT and NOCA, respectively. The $\beta$ values of strain prom*pfeA*-mCherry (filled blue circles, empty blue circles are outliers) are plotted according to the ENT concentration and those of strain prom*foxA*-mCherry (filled red circles, the empty red circles are outliers) according to the NOCA concentration. The sigmoid curve fitted with the Hill Eq. (1) on the prom*pfeA*-mCherry data is represented by the blue dashed line and the parameters obtained were: ${y}_{0}$ = 73.08, ${y}_{max}$ = 874.07, $n$ = 2.10, $K$ = 0.61 µM. The logarithmic model (2) based on prom*foxA*-mCherry data is represented by the red dashed line and the parameters obtained were: $a$ = 192.93 and ${x}_{E}$ = 0.45 µM. Outliers were not considered for either curve fitting.

Thus, within a factor of one, the gene activity can be deduced from the fluorescence curve after having fitted this fluorescence using Eq. (8). For certain conditions, in particular, conditions in which the fluorescence curve is close to zero, the fit of the synthesis Eq. (8) by the first-order model fails, leading to aberrant parameters (very-high ${P}_{\infty }$ and/or small $\tau$). The data of these conditions were removed for further analysis. The $\beta$ values of accepted conditions plotted for the strains prom*pfeA*-mCherry and prom*foxA*-mCherry are shown in Fig. 5. The rate of mCherry synthesis followed the gradient of ENT with a sigmoid shape for the prom*pfeA*-mCherry strain and in a logarithmic manner for the gradient of NOCA in the prom*foxA*–mCherry strain.

We treated the fluorescence data for prom*pfeA*-mCherry and prom*foxA*-mCherry as previously described to compare them to the RT-qPCR data. Again, we calculated the RMSE between the common model and the gene-specific model as a metric to assess the validity of this approach.

10 $$\widehat{{FC}_{RT-qPCR}} = \frac{{FC}_{RT-qPCR} -{y}_{0 RT-qPCR}}{{y}_{max RT-qPCR} -{y}_{0 RT-qPCR}},$$

11 $$\widehat{{FC}_{Fluo}} =\frac{{FC}_{Fluo} -{y}_{0 Fluo}}{{y}_{\mathrm{max}Fluo} -{y}_{0 Fluo}}.$$

**Figure 6:**
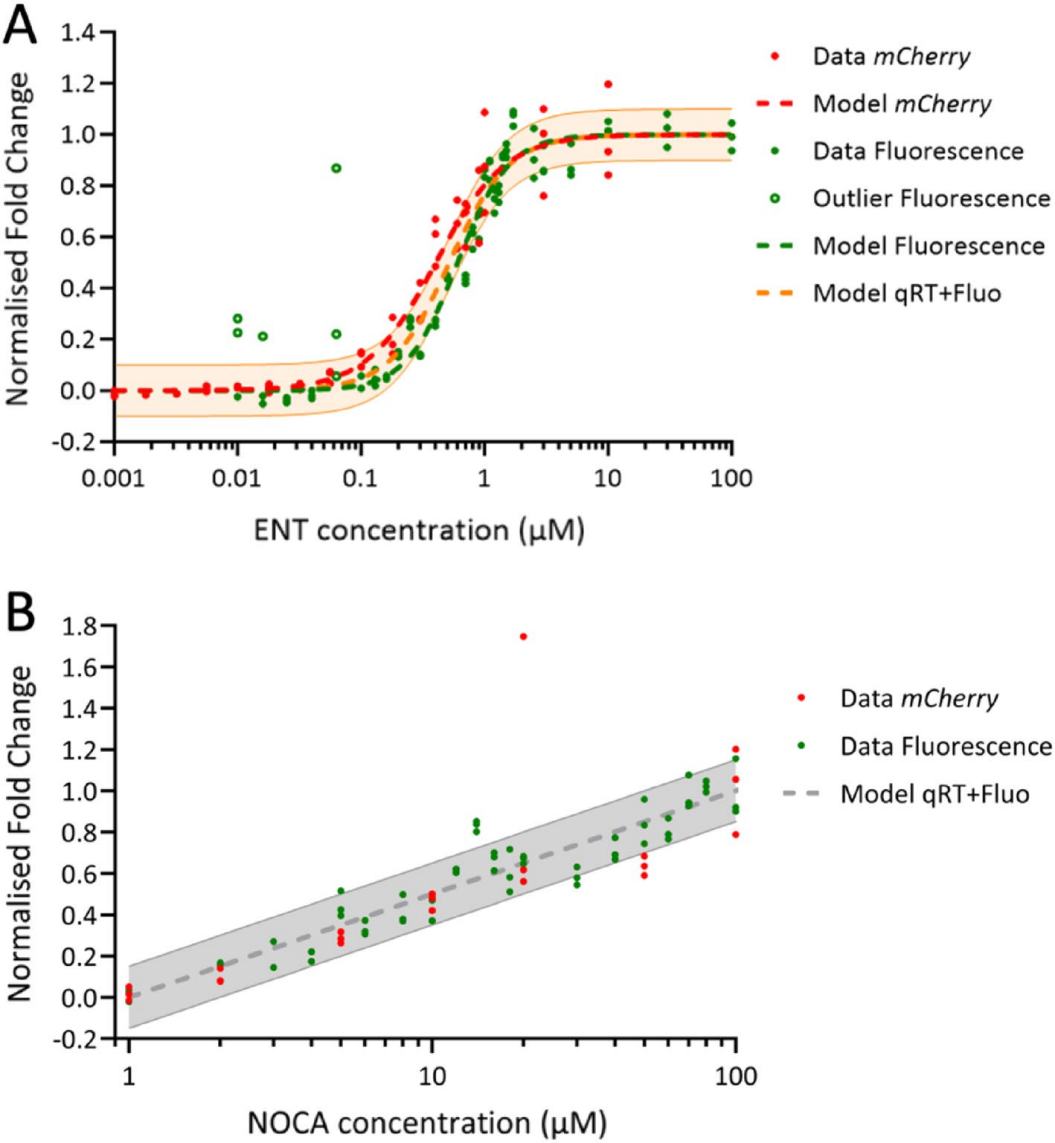
Normalization of the models from RT-qPCR data and fluorescence analysis. (**A**) The normalized set of RT-qPCR fold changes from prom*pfeA*-mCherry for *mCherry* are represented in red and the normalized set of $beta$ values from the fluorescence data in green (data as filled circles, outliers as empty circles, and the Hill curve as a dashed line). The Hill curve of *RT-qPCR* + *Fluo,* with an error of ± 10%, is represented as an orange dashed line with a transparent orange area. The table shows the Hill parameters of the different normalized data sets. (**B**) The normalized set of RT-qPCR fold changes from prom*foxA*-mCherry for *mCherry* is represented in red and the normalized set of $beta$ values from the fluorescence data in green. The normalized logarithmic model fitted for the three data sets is represented as a grey dashed line, with an error of ± 15% indicated by the transparent grey area.

The normalized and pooled data sets with the fitted models for prom*pfeA*-mCherry are represented in Fig. 6A. We performed a similar analysis as presented before and calculated the RMSE between the normalized *RT-qPCR* and *Fluo* whole data sets and the *RT-qPCR* + *Fluo* Hill equation. We found RMSE values of 0.068 and 0.107 for the comparison *RT-qPCR*/*RT-qPCR* + *Fluo* and *Fluo*/*RT-qPCR* + *Fluo*, respectively. We also calculated the RMSE for the transition phase, which involved first using only data between 0.1 and 0.9 and then 0.2 and 0.8 of the normalized fold change. The RMSE values were 0.083 and 0.099 for the data between 0.1 and 0.9 and 0.100 and 0.103 for the data between 0.2 and 0.8 for *RT-qPCR* and *Fluo* respectively. Thus, the error of our *RT-qPCR* + *Fluo* model is approximately 10%.

Applying a similar methodology as that used for the RT-qPCR data previously, we determined the inherent dispersion between the *RT-qPCR* or *Fluo* data and the *RT-qPCR* + *Fluo* model. The RMSE between the data and the model showed a comparable magnitude. The utilization of a single model for both datasets did not introduce any additional errors beyond the inherent variability in the data. Therefore, the validity of the model remains unaltered. Thus, the *RT-qPCR* and *Fluo* data, in other words, the transcriptional expression of *mCherry* and mCherry synthesis, appear to show analogous behavior in relation to the siderophore concentration.

As previously, for prom*foxA*-mCherry, both the transcriptional expression of *mCherry* and mCherry synthesis could be normalized and modeled by the logarithmic equation of *RT-qPCR* + *Flu*. The normalized models of the transcriptional expression of *mCherry* and mCherry synthesis and *RT-qPCR* + *Fluo* all had the same parameters ($a$ = 0.5 and ${x}_{E}$ = 1 µM, Fig. 6B). For the comparison of *RT-qPCR*/*RT-qPCR* + *Fluo*, the RMSE value was the same as that previously calculated, 0.151. We calculated the RMSE for the comparison of *Fluo*/*RT-qPCR* + *Fluo* and found a value of 0.087. Here, we also assessed the intrinsic dispersion between the *RT-qPCR* or *Fluo* data and the *RT-qPCR* + *Fluo* model. The RMSE between the data and the model were of similar magnitude. Employing a single model for both datasets did not introduce additional errors beyond the inherent variability. Thus, the model’s validity remains unchanged, indicating analogous behavior between the *RT-qPCR* and *Fluo* data in response to the siderophore concentration.

## Discussion

The use of mathematical equations allows the quantification and prediction of biological processes, which can provide insights that may not be immediately apparent from experimental data alone. For example, mathematical modeling can be used to make predictions about the relationships between different variables, such as the relationship between mRNA and protein levels. Additionally, mathematical models can also help to identify potential sources of error or uncertainty in the data and can be used to simulate different scenarios and test the robustness of a model. However, mathematical modeling usually requires large datasets. Here, we developed two biological constructs that are very helpful for the rapid and low-cost generation of datasets to monitor the expression of the two TBDTs FepA and FoxA in *P. aeruginosa* cells to predict the future expression of different TBDTs present in the genome of *P. aeruginosa*, depending on the bacterial environment.

First, the constructed fluorescent reporters were created by fusing the promoter region of the genes encoding the TBDTs PfeA and FoxA (Fig. 1) with the coding sequence of the fluorescent protein mCherry, used as reporter, to generate sufficient data for the mathematical modelling. These fusions were inserted into the genome of *P. aeruginosa* between the *glm*S and PA5548 genes, a region previously used for gene insertion^47^. With our prom*pfeA*-mCherry and prom*foxA*-mCherry constructs, an increase in fluorescent is expected when the expression of the gene encoding the TBDT is activated. Such constructs allow the screening of multiple conditions in 96-well microplates. RT-qPCR or proteomic approaches could have been used to follow the expression of genes encoding TBDTs^34–36,51^, but such approaches are much more costly and time consuming and it is impossible to rapidly generate a large amount of data.

We conducted various tests to verify that the prom*pfeA*-mCherry and prom*foxA*-mCherry constructs are reliable and that the mCherry fluorescence values are representative of the expression of the TBDT of interest. First, we used RT-qPCR to show that insertion of our sequence (TBDT promotor region with *mCherry*) in the genome of *P. aeruginosa* does not significantly modify the transcription of the *glm*S and *PA5548* genes and that the presence of two promoters in the genome (in front of the genes encoding the TBDT and mCherry) of *P. aeruginosa* does not interfere with the transcription of *pfeA* or *foxA*. We also verified that transcription of the gene encoding *mCherry* follows that of the gene encoding the studied TBDT for both constructs, demonstrating that *mCherry* transcription and expression are regulated by the promoter region of the TBDT of interest. RT-qPCR data showed parallel mRNA synthesis kinetics for the genes encoding mCherry and the TBDT of interest. However, we observed a higher level of transcription for the genes encoding PfeA or FoxA than *mCherry*, which was more pronounced for the fusion reporter carrying the *pfeA* promoter region. One possible reason is that the stability of the mRNA of *pfeA* or *foxA* and *mCherry* may differ, with the mRNA of *pfeA* or *foxA* being more stable, resulting in higher mRNA levels than for *mCherry*.

Overall, the controls we carried out show that the fluorescence of mCherry measured using our fluorescent reporters prom*pfeA*-mCherry and prom*foxA*-mCherry is representative of the expression of the transporters PfeA and FoxA, respectively. The use of these fluorescent reporters was highly successful and allowed us to generate a large amount of data to study the effect of increasing concentrations of siderophores.

Modelling the data generated using our two fluorescent reporters showed that the expression of the *pfeA* and *foxA* genes in the two strains, prom*pfeA*-mCherry and prom*foxA*-mCherry, does not respond in the same way or with the same efficiency to the presence of increasing concentrations of their corresponding siderophores. The transcription of *pfeA* showed a sigmoidal shape, whereas the transcription of *foxA* was logarithmic. Maximum transcription for *pfeA* was reached for 3 µM ENT and higher, whereas the maximum was not reached for *foxA* with 100 µM NOCA (Fig. 6). The Hill coefficient of the model suggests that the regulatory mechanism between *pfeA* and ENT is cooperative but that the transition between the expressed and inhibited states is smooth. The transition occurs just before 1 µM. Thus, the concentration of 10 µM ENT, which we generally used to activate *pfeA* transcription in previous publications^34^, is sensible and the behavior of the bacteria should not change much if the ENT concentration increases above 10 µM and should be stable for small variations of concentration around 10 µM.

On the other hand, the activation of *foxA* expression by NOCA was much weaker and we cannot consider that maximal expression is reached at 100 µM, which is our technical limit. At lower concentrations of NOCA, the changes in fluorescence were probably too small to be detected and at higher concentrations, NOCA started to precipitate. The logarithmic fitting used in this paper is only valid for the available range, but we have no idea of how the production rate evolves beyond 100 µM. Another consequence is that, in contrast to *pfeA*, the expression of *foxA* is not stable for concentrations around 10 µM and might change significantly, even for small variations of concentration around this value.

There are several possible explanations for the difference in the expression of *pfeA* and *foxA* as a function of the concentrations of their corresponding siderophores, the first being the different transcriptional regulatory systems involved. *pfeA* transcription is also regulated by two-component systems^27,45,46^, whereas sigma and anti-sigma factors are involved for *foxA*^30,35^. In the case of PfeA, the ENT-Fe complex has to bind to the PfeA binding site to be transported into the periplasm and interact in this cell compartment with the inner membrane sensor PfeS of the transcriptional regulatory system^27^. This PfeS-ENT-Fe interaction then liberates the transcriptional regulator PfeR to activate transcription of the *pfeA* gene. Nothing is known about the affinity of PfeS for ENT-Fe or the mechanism of interaction. Likely, on this transcriptional regulation of *pfeA* by the PfeS/PfeR couple, another regulation is probably integrated involving two other two-component systems, PirS/PirR and CzcS/CzcR^46^. The first is implicated in the transcriptional regulation of *pirA*, a TBDT involved in the import of iron, either by ENT or monocathechol-type siderophores^32,37,52^. The second system, CzcS/CzcR, is involved in Zinc homeostasis, heavy metal and antibiotic resistance and swimming motility^53–55^. In the case of *foxA* transcription, the NOCA-Fe complex has to interact with the FoxA binding site at the cell surface to obtain an interaction between the signaling domain of the TBDT and its anti-sigma factor FoxI^56^. This protein interaction leads to dissociation of the transcriptional regulator FoxR from FoxI, which can then interact with the promoter region of *foxA* to drive the transcription of this gene. In this mechanism, the binding of NOCA-Fe to the PfeA binding site is a key step to induce the system. The regulation mechanisms of the transcription of *pfeA* or *foxA* are very different and of different complexity are may not function with the same efficiency. They involve different protein–protein and siderophore-protein interactions and in the case of ENT-Fe, its uptake into the periplasm.

There are other factors upstream and downstream of these two regulatory cascades that can affect the efficiency of *pfeA* and *foxA* gene transcription. These include the affinity of the two siderophores for ferric iron, with ENT having a higher affinity than NOCA at neutral pH: Ka = 10^49^ M^−1^ for ENT and Ka = 10^32^ M^−1^ for NOCA^38,39^. Consequently, ENT will be more efficient for the competition for iron with pyoverdine and pyochelin (the two siderophores produced by *P. aeruginosa*) than NOCA.

In addition, when the siderophore-Fe complexes are formed, NOCA-Fe binds to its binding site on FoxA, located on the plug domain of the FoxA structure, with a Kd of 178 nM before being imported through the outer membrane^16,35^. Unlike the NOCA-Fe/FoxA pair, ENT-Fe can interact with two binding sites on PfeA, one located in the extracellular loops and the second on the plug domain of PfeA^15^. Affinity measurements showed that one of these sites is a high affinity site and the other a lower affinity site (Kd = 60 nm and Kd = 155 µM for the two binding sites on PfeA)^15^. Currently it is impossible to know which of the two sites identified by crystallography is the low or high affinity site. The differences between the binding of FoxA and PfeA to their siderophore-Fe complexes may affect the regulation of the transcription of the *foxA* and *pfeA* genes differently, as this binding step is essential in the regulatory process in both cases. Furthermore, in the cytoplasm, the promoter regions of the *pfeA* and *foxA* genes are different and the mechanisms of recognition of PfeR and FoxI are different and may also affect the efficiency of transcription.

The systems and mechanisms of transcriptional regulation of the genes involved in the two iron import pathways are different and highly complex, involving different protein–protein interactions and different interactions between proteins and the siderophore-Fe complexes. The mathematical model described in this paper is more a phenomenological model than a precise description of the biochemical mechanisms involved. The building of such a model would obviously be an asset in understanding regulatory phenomena but would require the parallel measurement of various concentrations of several molecules, which is not straightforward from an experimental point of view. Model refinement techniques could make it possible to generate hypotheses, but these can be very difficult to validate experimentally. Due to such complexity, it is currently impossible to identify which factor(s) is/are responsible for the large differences in the expression kinetics of the *foxA* and *pfeA* genes as a function of the concentration of their respective siderophores. As a next step, it would be informative to test other siderophores (analogues of ENT and NOCA) that can be recognized by these two TBDTs to determine whether they affect the expression of these two genes in the same way.

It would also be informative to test mixtures of different concentrations of ENT and NOCA and see the effect on the expression of these two genes in a situation in which both siderophores are in competition for iron. The tools that we have developed here can also help improve our understanding of the regulatory mechanisms involved in the expression of TBDTs. One can imagine, for example, testing deletions of genes encoding transcriptional regulators, such as PfeS/PfeR or FoxI/FoxR, or amino acid mutations of the binding sites of PfeA or FoxA and assessing the effect on our model.

In conclusion, the promoter fusions prom*pfeA*-mCherry and prom*foxA*-mCherry are powerful tools to follow the expression of a TBDT in *P. aeruginosa*. Mathematical modelling of the data obtained showed that the expression of *pfeA* displays a sigmoidal shape, whereas it is logarithmic for *foxA*, with maximum transcription for *pfeA* at 3 µM ENT, whereas the maximum was not reached with 100 µM NOCA for *foxA*. These results have important implications for the understanding of how *P. aeruginosa* adapts the expression of its TBDTs to changing environmental conditions and highlight the value of using mathematical models as a tool to investigate iron homeostasis and, more generally, bacterial physiology. Our results provide a foundation for the development of a larger system for studying the regulation of all iron acquisition pathways of *P. aeruginosa*.

## Materials and methods

### Chemicals

Enterobactin (ENT) was purchased from Sigma-Aldrich and Nocardamine (NOCA) was purified as previously described^57^.

### Bacterial strains and growth conditions

*P. aeruginosa* strains used in this study are listed in Table S1 in Supplemental Materials. Bacteria were first grown in Lysogeny Broth (LB) at 30 °C overnight. Afterwards, they were pellet, washed and resuspended in iron-deficient CAA (casamino acid) medium (composition: 5 g L^−1^ low-iron CAA (Difco), 1.46 g L^−1^ K_2_HPO_4_ 3H_2_O, 0.25 g L^−1^ MgSO_4_ 7H_2_O) and grown over night at 30 °C. In order to monitor growth in the presence of siderophores, cells were resuspended again in fresh CAA medium at an optical density at 600 nm (OD_600 nm_) of 0.01, grown in 96 well plates, in the absence or presence of increasing concentrations of ENT (0.001–100 µM) or NOCA (0.01–100 µM). Plates were incubated at 30 °C, with shaking every 15 min, in a microplate reader (Infinite® 200 PRO M Nano+, Tecan). Growth was followed by measuring the OD_600 nm_ and the fluorescence of mCherry (excitation at 570 nm and emission at 610 nm) every 30 min, for 24 h.

### Construction of the prompfeA–mCherry and promfoxA–mCherry strains

*Escherichia coli* TOP10 (Invitrogen) was used as the host strain for the plasmids. The DNA fragments from *P. aeruginosa* used for cloning were amplified from the genomic DNA of strain PAO1 with Phusion High-Fidelity DNA polymerase (Thermo-Fisher Scientific). The insertion of the sequence was done in the intergenic region between *glmS* and *PA5548* using a pEXG2 plasmid^58^. The construction of the plasmids was realized with the NEBuilder® HiFi DNA Assembly Master Mix. The primers are listed in Table S2 in Supplemental Materials. As previously described, recombinant clones were isolated and then verified by PCR and Sanger sequencing^37^.

### Quantitative real-time PCR analyses

Specific gene transcription was measured by reverse transcription quantitative PCR (RT-qPCR). Overnight cultures of bacterial cells grown in CAA medium were pelleted, resuspended and diluted in fresh medium to obtain an OD_600 nm_ of 0.1. The cells were then grown with or without siderophores, under shaking, at 30 °C for 8 h. Afterward, 2.5 × 10^8^ cells were mixed with two volumes of RNAprotect Bacteria Reagent (Qiagen). Samples were lysed in Tris–EDTA at pH 8.0 containing 15 mg mL^−1^ lysozyme (Sigma-Aldrich) for 15 min at 25 °C. Afterwards, total RNA was extracted using an RNeasy MinElute Spin Columns and gDNA Eliminator Spin Columns from RNeasy Plus Micro Kit (Quiagen). A treatment by DNase (RNase-Free DNase Set, Qiagen) was realized for each sample Reverse transcription of RNA was done using the iScript™ cDNA Synthesis Kit (Bio-Rad) with 1 µg of input RNA. Gene expression was measured with a CFX Opus 96 Real-Time PCR Instrument (Bio-Rad) using iTaq Universal SYBR Green Supermix (Bio-Rad) and the appropriate primers (listed in Supporting Information Table S2). Five genes (*rpoD*, *clpX*, *rpsL*, *proC* and *uvrD*) were tested as reference genes using the Reference Gene Selector Tool based on GeNorm in CFX Maestro™ Software (Bio-Rad). PAO1, prom*pfeA-*mCherry and prom*foxA*-mCherry strains were grown for 8 h in the absence and presence of siderophores (10 µM ENT for PAO1 and prom*pfeA-*mCherry, 100 µM of NOCA for PAO1 and prom*foxA*-mCherry). We have chosen to use *clpX* and *rpoD* in our assays because their stability across the different conditions were the highest (Fig. S4). For the data analysis, the quantification cycles were determined by regression and the fold-change calculated with the Pfaffl Method.

### Data processing and mathematical modeling

All the data processing and the mathematical modeling was done with Python 3.8 (https://www.python.org/) and packages (https://pypi.org/): Pandas was used to manipulate and manage the data; NumPy and SciPy were used to process the data and create the models; Matplotlib was used for visualizations. The equation parameters were found by using the curvefit function from scipy.optimize on our experimental data.

### Supplementary Information

Supplementary Information.

## Data Availability

All generated data are included in this manuscript and Supplementary Files.
