# Supplementary material for: Experimental and computational methods to highlight behavioural variations in TonB-dependent transporter expression in Pseudomonas aeruginosa versus siderophore concentration
Source: Sci Rep. 2023 Nov 16;13:20015. doi: 10.1038/s41598-023-46585-z (PMC10654771; doi:10.1038/s41598-023-46585-z)
Supplement: Supplementary file 1 — Supplementary Information. [file 41598_2023_46585_MOESM1_ESM.pdf]

Supplementary Materials

Experimental and computational methods to highlight behavioural variations in TonB-dependent transporter expression in *Pseudomonas aeruginosa* versus siderophore concentration

Thibaut Hubert[1,2,3], Morgan Madec[3*], Isabelle J. Schalk[1,2*]

Table S1. Strains and plasmids used in this study

Strains and plasmids	Collection ID	Relevant characteristics	References
P. aeruginosa			
PAO1	PAO1	*P. aeruginosa* wild-type strain	1
prom*pfeA*-mCherry	PAS1111	PAO1 with the promoter sequence of *pfeA* followed by *mcherry* inserted in the genome	This study
prom*foxA*-mCherry	PAS1112	PAO1 with the promoter sequence of *foxA* followed by *mcherry* inserted in the genome	This study
E. coli			
TOP10		*F- mcrA Δ(mrr-hsdRMS-mcrBC) φ80lacZΔM15 ΔlacX74 nupG recA1 araD139 Δ(ara-leu)7697 galE15 galK16 rpsL(Str^R) endA1 λ*	Invitrogen
**Plasmids**			
pTH1	551	pEXG2 carrying the promoter sequence of *pfeA* followed by the sequence of mCherry	This study
pTH2	552	pEXG2 carrying the promoter sequence of *foxA* followed by the sequence of mCherry	This study

Table S2. Oligonucleotides used in this study

Oligonucleotides	Sequences (5’ to 3’)	Use
ppfeA F	CGTAACTAGTCCTCCCCTACCAGCGCCT	Construction plasmid pTH1
ppfeA R	TTCTAGACATCGGTGATCTCCGGGCAATG	Construction plasmid pTH1
pEXG2 *ppfeA* F	GAGATCACCGATGTCTAGAAGCAAGGGC	Construction plasmid pTH1
pEXG2 *ppfeA* R	GTAGGGGAGGACTAGTTACGTGGCCTGTAG	Construction plasmid pTH1
pfoxA F	CGTAACTAGTCGGGGTCTATCGCCTGGAC	Construction plasmid pTH2
pfoxA R	TTCTAGACATGAACGGGATCCGTTGGAG	Construction plasmid pTH2
pEXG2 *pfoxA* F	GATCCCGTTCATGTCTAGAAGCAAGGGC	Construction plasmid pTH2
pEXG2 *pfoxA* R	ATAGACCCCGACTAGTTACGTGGCCTGTAG	Construction plasmid pTH2
clpX F	CTGCGCTCATGCAGATCCT	RT-qPCR
clpX R	TCGAACAGCTTGGCGTACTG	RT-qPCR
proC F	GGCGTATTTCTTCCTGCTGA	RT-qPCR
proC R	TGGCCTGGAAGGATTTGAT	RT-qPCR
rpoD F	ACAAGATCCGCAAGGTACTGAAG	RT-qPCR
rpoD R	CGCCCAGGTGCGAATC	RT-qPCR
rpsL F	TACACCACCACGCCGAAAA	RT-qPCR
rpsL R	CACCACCGATGTACGAGGAA	RT-qPCR
uvrD F	CTACGGTAGCGAGACCTACAACAA	RT-qPCR
uvrD R	GCGGCTGACGGTATTGGA	RT-qPCR
mcherry F	CAACGTCAACATCAAGTTGGACAT	RT-qPCR
mcherry R	CGTACTGTTCCACGATGGTGTAG	RT-qPCR
pfeA F	GCCGAGACCAGCGTGAAC	RT-qPCR
pfeA R	GGCCGGATTCGATCTTGTT	RT-qPCR
foxA F	AAGGGCTCGGATACCCAGTT	RT-qPCR
foxA R	CGTTGGGATCGTGTTGCA	RT-qPCR

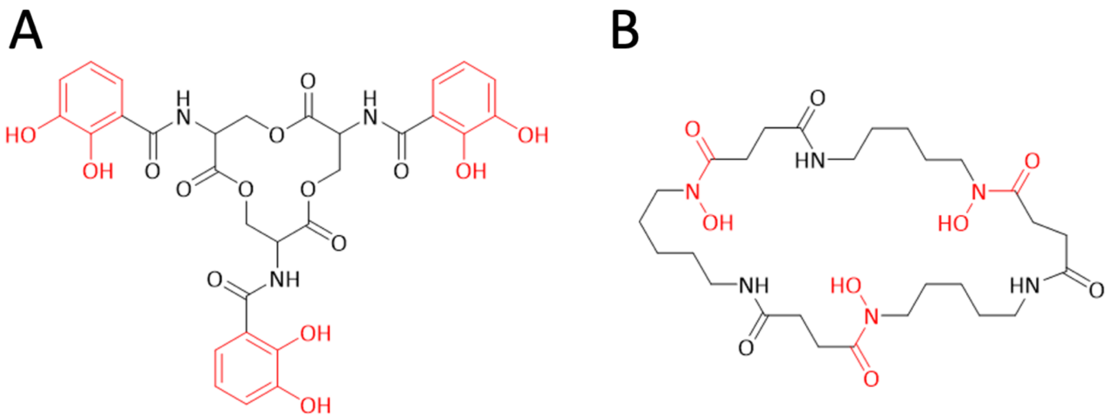

Figure S1. Structure of enterobactin (ENT) (A) and nocardamine (NOCA) (B). Chelating functions are shown in red.

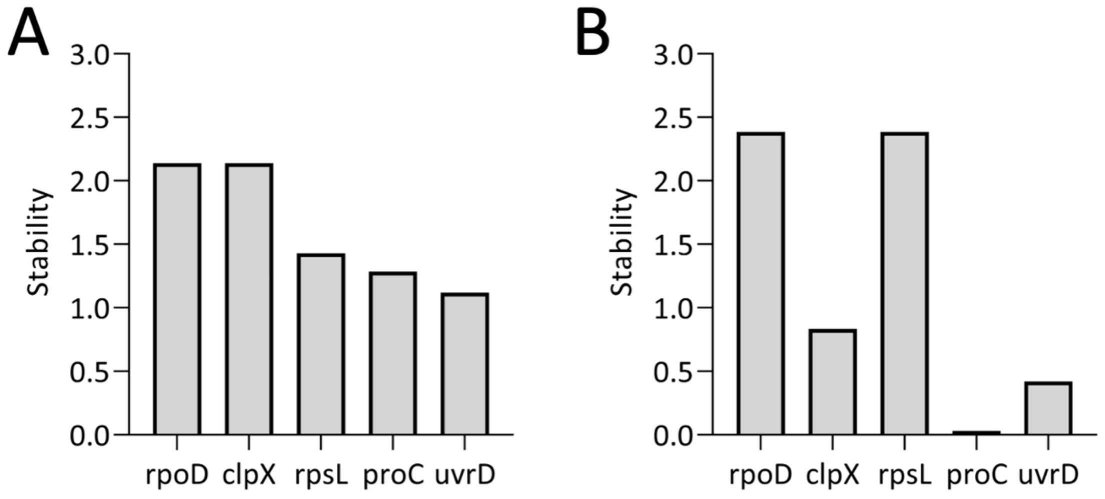

Figure S2. Reference gene stability plot. prom*pfeA*-mCherry was grown in the absence or presence of 0.1, 1, or 10 µM ENT (A) and prom*foxA*-mCherry in the absence or presence of 1, 10, or 100 µM NOCA. In all cultures, the transcription of *rpoD*, *clpX*, *rpsL*, *proC*, and *uvrD* was followed by RT-qPCR. Previous studies have shown the expression of these genes to be stable in *P. aeruginosa*.[2–6] A gene can be considered as a reference gene for the experiments when its stability over the conditions tested is above 0.5. The results are expressed as the ratio of the values obtained for prom*pfeA*-mCherry and prom*foxA*-mCherry grown in the absence or presence of the siderophores. Biological triplicates were carried out and the data were analyzed as described by Vandesompele *et al.*[7] *rpoD* and *clpX* were selected as reference genes for all RT-qPCR analyses presented in this study.

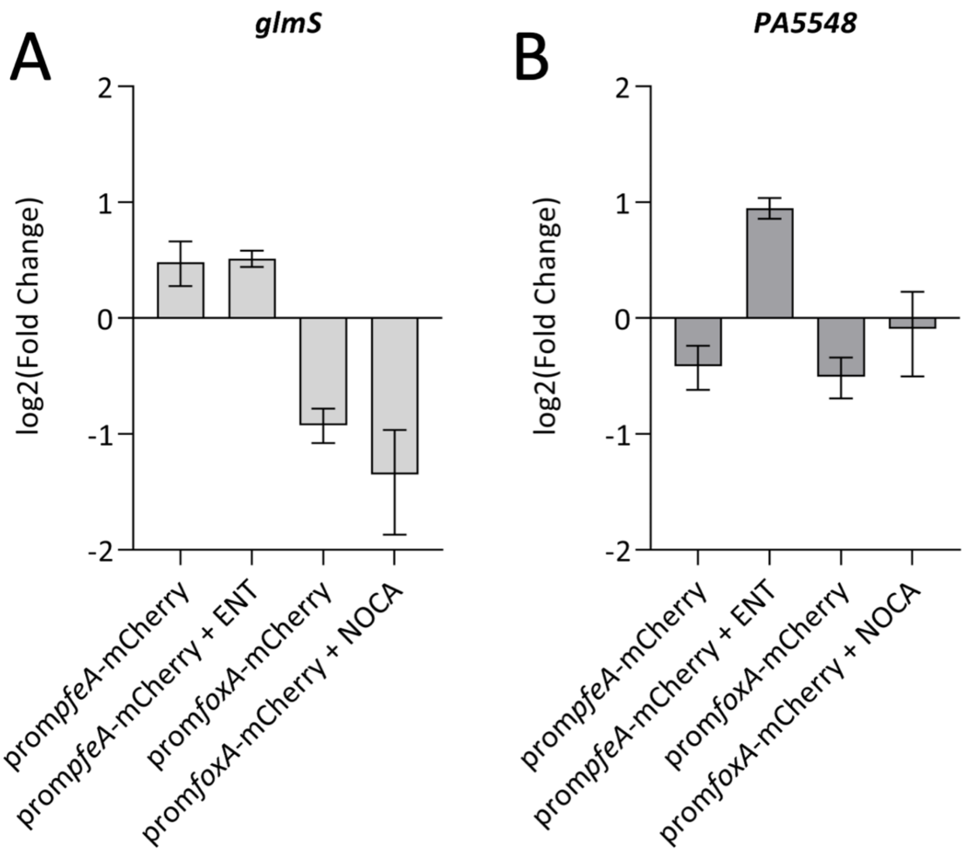

Figure S3. RT-qPCR on *glmS* (A) and *PA5548* (B) genes. PAO1 was grown for 8 h in the absence of siderophores. prom*pfeA*-mCherry and prom*foxA*-mCherry strains were grown for 8 h in the absence or presence of 10 µM ENT or 100 µM NOCA, respectively. In all cultures, the transcription of *glms* and *PA5548* was followed by RT-qPCR. The results are expressed as the ratio of the values obtained for prom*pfeA*-mCherry and prom*foxA*-mCherry grown in the absence or presence of the siderophores compared to those of PAO1 (grown in the absence of siderophores). Biological triplicates were carried out. The error bars represent the standard deviations calculated by CFX Maestro™ Software (Bio-Rad). The reference genes used were *clpX* and *rpoD*.

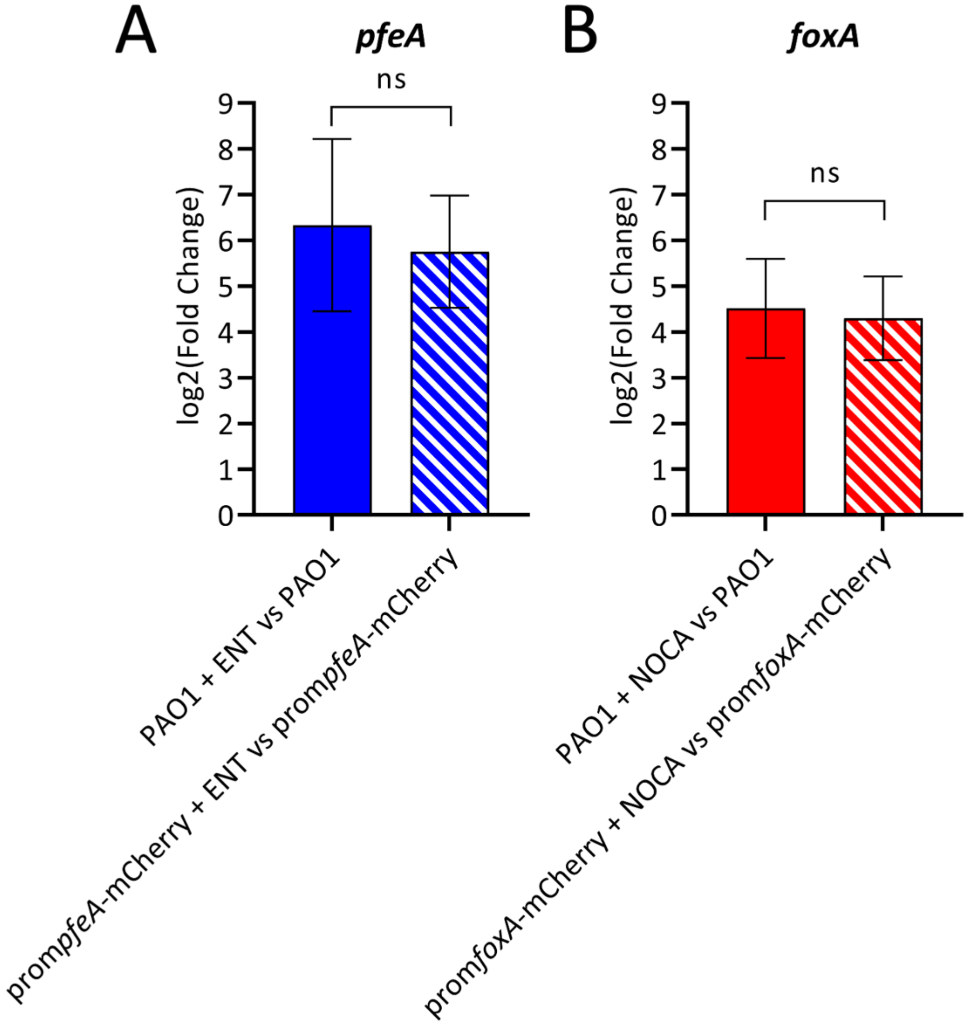

Figure S4. RT-qPCR to verify whether the insertion in the genome of a second promoter modifies the transcription levels of *pfeA* and *foxA*. (A) PAO1 and prom*pfeA*-mCherry strains were grown for 8 h in the absence or presence of 10 µM ENT. The transcription of *mCherry* and *pfeA* was followed by RT-qPCR. (B) PAO1 and prom*foxA*-mCherry were grown in the presence of 100 µM NOCA. The transcription of *mCherry* and *foxA* was followed by RT-qPCR. The results are expressed as the ratio of values obtained with the siderophores to those obtained without for both assays. Biological triplicates were carried out. The error bars represent the standard deviations calculated by CFX Maestro™ Software (Bio-Rad). The reference genes used were *clpX* and *rpoD*.

References:

1. Stover, C. K. *et al.* Complete genome sequence of *Pseudomonas aeruginosa* PAO1, an opportunistic pathogen. *Nature* **406**, 959–64 (2000).
2. Savli, H. *et al.* Expression stability of six housekeeping genes: A proposal for resistance gene quantification studies of *Pseudomonas aeruginosa* by real-time quantitative RT-PCR. *J Med Microbiol* **52**, 403–408 (2003).
3. Konings, A. F. *et al. Pseudomonas aeruginosa* uses multiple pathways to acquire iron during chronic infection in cystic fibrosis lungs. *Infect Immun* **81**, 2697–2704 (2013).
4. Alqarni, B., Colley, B., Klebensberger, J., McDougald, D. & Rice, S. A. Expression stability of 13 housekeeping genes during carbon starvation of *Pseudomonas aeruginosa*. *J Microbiol Methods* **127**, 182–187 (2016).
5. Ahmed, S. A. K. S. *et al.* Natural quorum sensing inhibitors effectively downregulate gene expression of *Pseudomonas aeruginosa* virulence factors. *Appl Microbiol Biotechnol* **103**, 3521–3535 (2019).
6. Perraud, Q. *et al.* Phenotypic Adaption of *Pseudomonas aeruginosa* by Hacking Siderophores Produced by Other Microorganisms. *Mol. Cell Proteomics* **19**, 589–607 (2020).
7. Vandesompele, J. *et al.* Accurate normalization of real-time quantitative RT-PCR data by geometric averaging of multiple internal control genes. *Genome Biology* **3**, research0034.1 (2002).
